# Health Indicators of Pregnant Women in Tonkolili District, Rural Sierra Leone

**DOI:** 10.3390/ijerph17113918

**Published:** 2020-06-01

**Authors:** Daphne Bussink-Voorend, Anton P. Bussink, Abdul M. Falama, Jelle Stekelenburg

**Affiliations:** 1 Lion Heart Medical Centre, Yele, Gbonkolenken, Tonkolili District, Sierra Leone; 2 Lion Heart Foundation INGO, Yele, Gbonkolenken, Tonkolili District, Sierra Leone; apbussink@gmail.com; 3 District Health Management Team Tonkolili, Primary Health Complex, Magburaka, Tonkolili District, Sierra Leone; abdulmac14@yahoo.com; 4 Department of Health Sciences, Global Health, University Medical Centre Groningen, 9700 AD Groningen, The Netherlands; jelle.stekelenburg@online.nl; 5 Department of Obstetrics and Gynaecology, Leeuwarden Medical Centre, 8934 AD Leeuwarden, The Netherlands

**Keywords:** antenatal care, Sierra Leone, malaria in pregnancy, maternal malnutrition, anaemia in pregnancy, adolescent pregnancy

## Abstract

Despite having reported one of the highest maternal mortality ratios and neonatal mortality rates in the world, surprisingly little is known about the general health status of pregnant women in rural parts of Sierra Leone. Malaria, anaemia and malnutrition are known contributors to adverse pregnancy outcomes. Although their prevalence is known to be high, the burden of these conditions in the rural pregnant population remains unknown. Our study aimed to gain more insight into the health status of pregnant women. An observational retrospective descriptive study was conducted at the Lion Heart Medical Centre using antenatal care (ANC) registers. The study revealed high prevalence of malaria (35.2%), maternal undernutrition (10.4%) and anaemia (65.9%). The proportion of teenage pregnancies in the ANC population was 16.4%. Both malaria and anaemia were more prevalent in this group, with odds ratios of 2.1 and 1.7, respectively. The findings reveal alarming high rates of anaemia, acute undernutrition and malaria among pregnant women and high numbers of pregnancy among adolescents, with increased health risks. These results will be used to advocate for a malnutrition program, specifically for pregnant women. Our study further emphasises the importance of preventing malaria and anaemia in pregnant women.

## 1. Introduction

Sierra Leone has one of the highest maternal mortality ratios (MMR) in the world according to the latest estimates by the World Health Organization (WHO), but surprisingly little is known about the health status of pregnant women in the Sierra Leonean setting [1]. The information that is available mainly comes from the demographic health survey (DHS) conducted in 2013 and the preliminary key findings from the DHS conducted in 2019. The main findings regarding pregnancy and childbirth include high numbers of teenage pregnancies, high rates of anaemia and malaria among pregnant women and a trend of increasing institutional deliveries [2,3]. For the entire Sub-Saharan African region, neonatal mortality has decreased substantially in the past decades, and the most recent estimate for 2017 is 27 deaths per 1000 live births [4]. Reports specifically for Sierra Leone indicate that neonatal mortality rate was 35 per 1000 live births in 2015, considerably higher than the regional average [5]. The recent DHS report indicates that neonatal mortality has dropped to 31 per 1000 live births [3].

Malaria infection during pregnancy is a well-known cause of impaired foetal growth due to disturbed development of the placenta, leading to low birth weight (LBW) [6,7,8]. An infection during the second trimester has the most negative effect on birth weight due to a disturbed development of the placenta [6]. In malaria endemic areas such as Sierra Leone, pregnant women are more susceptible to be infected than non-pregnant individuals. This susceptibility reduces in subsequent pregnancies, and therefore primigravid women bear the highest risk. At the same time, it may be challenging to diagnose malaria during pregnancy in areas with high transmission, since *Plasmodium falciparum* is often undetectable in blood smears due to sequestration of the parasite in the placenta. Additionally, in endemic areas, infections are often asymptomatic, leading to chronic infections in pregnant women, which contribute to the development of anaemia and LBW [9]. Estimates indicate that, in malaria endemic areas like Sierra Leone, 50% of LBW in infants can be attributed to malaria [10]. Strategies endorsed by the WHO to reduce malaria infections during pregnancy include the widespread dissemination of bed nets, intermittent preventive treatment with Sulfadoxine/Pyrimethamine (IPTp-SP) and adequate case management of malaria infections in pregnancy.

Low birth weight is an important indirect cause of neonatal deaths, in particular for infants suffering from birth asphyxia and neonatal sepsis. Together, these factors are responsible for 60% of all neonatal deaths [11]. In addition to malaria, other factors that put infants at risk for low birth weight include maternal undernutrition and maternal iron-deficiency anaemia [12,13].

Mild anaemia during pregnancy is associated with an increased risk of stillbirths, neonatal deaths and LBW infants. These risks increase further when anaemia is moderate or severe [13]. Anaemia is mainly caused by dietary deficiencies, predominantly a lack of iron. Infections such as malaria, HIV and soil-transmitted helminths also contribute to or aggravate anaemia [14].

Maternal malnutrition is a serious underlying cause of adverse birth outcomes for both mother and child. Undernutrition during pregnancy increases the risk of an LBW infant and preterm delivery [12]. Scientific evidence on the relationship between undernutrition and maternal deaths is limited, but one study found an increased MMR in women with low mid-upper arm circumference (MUAC) measurement [15]. Other reports state that undernutrition together with anaemia is responsible for 20% of all maternal deaths at delivery [11].

In light of the high maternal mortality ratio and high neonatal mortality rate, it can be suspected that the burden of malaria, anaemia and malnutrition is substantial. So far, data regarding these health indicators are only available from the DHS.

An analysis of existing ANC services in Sierra Leone showed that the lack of essential supplies and infrastructure at service delivery points combined with late first ANC visits is the main hurdle towards the provision of adequate ANC services [5,16]. Benefits that could result from ANC services are missed; these include prevention of mother-to-child transmission (PMTCT) of HIV, anaemia treatment and intermittent preventive treatment in pregnancy with IPTp-SP to combat malaria.

In Yele, Tonkolili district, the Lion Heart Medical Centre (LHMC) has implemented an ongoing program to complement and strengthen the provision of ANC services. This program, known as the Healthy Baby Voucher program (HBVP), involves a full screening of pregnant women once in their pregnancy to identify and follow up high-risk cases. In order to get an accurate insight into the presence of health indicators that contribute to adverse birth outcomes and the characteristics of women utilizing ANC services through this program, a retrospective survey was conducted. The overall aim of the survey was to document the health indicators of pregnant women in Tonkolili district, Sierra Leone.

## 2. Materials and Methods

The study took place at the LHMC, a 70-bed referral hospital located in Tonkolili district, Gbonkolenken chiefdom. Surrounded by 15 primary healthcare units, the direct catchment area of the hospital consists of approximately 100,000 inhabitants. Pregnant women receive routine ANC from their nearest health centre and are invited to visit the LHMC for additional screening in the 2nd trimester. This service is covered by HBVP, funded by the Dutch Lion Heart Foundation, which aims to increase coverage and quality of ANC services by identifying and following up high risk cases, whereas uncomplicated cases are referred back to the nearest primary healthcare unit (PHU). The screening consists of anthropometric measurements, history taking, physical examination, laboratory investigations and ultrasound imaging. The HBVP includes provision for transport from and back to the PHU, and all services are performed free of charge.

A retrospective analysis of the ANC records was conducted with the aim to map baseline characteristics and determine the prevalence of maternal malnutrition, adolescent pregnancies, malaria, HIV, syphilis and anaemia in pregnant woman. Approval for the study was obtained from the Sierra Leone Ethics and Scientific Review Committee. Additionally, the board of the LHMC gave institutional approval. Since the study involved a retrospective analysis of existing records, patient consent was not obtained.

The desired sample size was calculated using Cochran formula [17] (Equation (1)).

*n* = *Z2p* × (*p* − 1)/*e2* (1)

When a confidence interval (*Z*) of 95% is used to estimate the precision (*e*), the sample sized is calculated using expected prevalence (*p*), indicated by findings from the DHS [2]. The analysis showed that a sample size of 380 or more would give statistically significant results for malaria, anaemia and malnutrition, with precision rates (e) of 5%, 5% and 1%, respectively. Subsequently, a desired sample size of 500 first ANC visits was chosen. A time frame of 5 months was selected, and all data between August and December 2018 were collected.

Data were extracted from existing ANC records by the corresponding author and transferred into a digital database (Microsoft Excel^®^, Microsoft, Redmond, WA, USA). The second author checked data entry. Each visit was encoded using a study number, personal data were not included to ensure privacy. The following information was recorded in the database: age, gravidity, parity, height, weight, MUAC, estimated gestational age (EGA) by ultrasound findings. Furthermore, laboratory results were matched with ANC visits and included in the database: haemoglobin (Hb) level, HIV, syphilis and rapid diagnostic test (RDT) result for infection with *P. falciparum*.

The Hb levels in the study population were routinely checked using the HemoCue^®^ 301 (HemoCue AB, Angelholm, Sweden). All women were screened for malaria, irrespective of complaints, with an RDT (CareStart™ Malaria HRP2, Access Bio, Somerset, NJ, USA). During pregnancy, the use of RDTs is preferred over light microscopy due to the possibly diminished peripheral parasite density caused by sequestration of the parasites in the placenta [16]. The SD Bioline HIV/Syphilis duo test (Standard Diagnostics, Gheung-gu, Republic of Korea) was used routinely to detect antibodies against HIV-1/2 and *Treponema pallidum*.

MUAC was routinely measured for all women with an adult MUAC tape. The MUAC is rather insensitive with respect to the physical changes during pregnancy and therefore preferred [18,19]. According to international consensus, undernutrition (body mass index (BMI) < 18.5) in pregnancy correlates with MUAC < 23 cm in adults, obesity (BMI > 30) is reflected in a MUAC reading >30 cm [18]. The women in our study were grouped according to their nutritional status. Since these cut-off values are only validated for adults, adolescents and clients without a recorded age were excluded from further analysis. Only data from first antenatal visits were included in further analysis to prevent double counting and overrepresentation of complicated cases that would come for follow-up visits.

### Statistical Analysis

For statistical analysis, the data were imported into an SPSS database (IBM^®^ SPSS^®^ statistics 25, Chicago, IL, USA). Prevalence values were determined using the frequencies analysis function for the entire study population as well as relevant subgroups (adolescents versus adults; primipara versus multipara; under-nourished versus well nourished). To allow further comparison between subgroups, the odds ratio (OR) was calculated by comparing the probability of malaria and anaemia (outcome) between subgroups with different characteristics (exposure). The 95% confidence interval (CI) was calculated to estimate the precision of the odds ratio, and when the CI did not overlap with the null value (OR = 1), statistical significance was assumed [20,21,22].

In the occasion of any missing values, the case was excluded for that particular analysis.

## 3. Results

Between August and December 2018, a total of 865 women visited the ANC clinic, of which 611 (71%) were at their first visit. Women who presented at our ANC clinic were on average 26 years old, varying from 12 to 54 years. It should be noted that the records were based on self-reported age, and outliers were seen at the round numbers 20, 25, 30 and 35 years (Table 1).

### 3.1. Health Indicators

The records for the entire group of 611 first ANC visits were analysed and screened for the presence of health indicators, as described. An overview of the results is shown in Table 2.

### 3.2. Anaemia

Hb levels were available for 551 women (90.2%). Anaemia was defined according to the WHO guidelines and grouped into mild (Hb 10.0–10.9 g/dL), moderate (Hb 7.0–9.9 g/dL) and severe (Hb < 7.0 g/dL) for pregnant women [23]. A total of 65.9% of all pregnant women were anaemic, and 34.1% presented with a normal haemoglobin level of 11 g/dL or higher. As shown in Table 2, 30.1% of pregnant women were diagnosed with mild anaemia, and 35.6% with moderate anaemia. Severe anaemia was seen in only one case, representing 0.2% of the study population.

### 3.3. Infections during Pregnancy: HIV, Syphilis and Malaria

Regarding HIV, test results were available for 495 women (80.4%). As indicated in Table 2, the incidence of HIV in our study population was 0.8%. The incidence of syphilis in pregnancy was 0.2%. During the entire study period, a positivity rate for malaria of 35.2% was recorded. Due to seasonal variations, the highest positivity rate was found in August (48.1%), and the lowest one in December (21.5%), as shown in Table 3. Pregnant women were routinely tested for malaria, irrespective of complaints. Therefore, the group of women with a positive test includes asymptomatic, mild and severe cases. When comparing primipara to multipara, malaria was more prevalent in primipara, with an odds ratio (OR) of 1.6 (CI 1.064–2.572).

### 3.4. Nutrition Status

MUAC values of adult pregnant women were analysed, as shown in Table 2. The results show that 10.4% of the participants were classified as undernourished, and 5.9% as obese. Further analysis was carried out to find if anaemia and malaria were more common in undernourished compared to well-nourished women. The ORs were, respectively, 1.7 (CI 0.8687–3.229) and 0.9 (0.4431–1.768) and were both non-significant.

### 3.5. Adolescents

Among pregnant women attending their first ANC visit, 16.4% were aged between 10 and 19 years and therefore classified as adolescent pregnancies according to the WHO definition. Among adolescents, 42 (50.6%) out of 83 were positive for malaria at their first ANC visit, which was higher compared to adults, for whom 143 (32.4%) positive cases out of 442 were recorded. The odds ratio for malaria in adolescents compared to adults was 2.1 (CI 1.335–3.441). For anaemia, 68 (75.6%) out of 90 pregnant adolescents were anaemic compared to 295 (64.0%) out of 461 adults. The OR for anaemia was 1.7 (CI 1.045–2.884).

## 4. Discussion

The present study is an attempt to document and characterize the health status of pregnant women in rural Sierra Leone, quantified by health indicators among the beneficiaries of our HBVP. The HBVP aims to improve the quality of ANC services. In our view, the most remarkable results are the high prevalence of malaria (35.2%), maternal undernutrition (10.4%) and anaemia (65.9%) among the pregnant population attending ANC for the first time in the LHMC.

In our study, 35.2% of all pregnant women had a positive RDT when screened, regardless of the presence of symptoms. Malaria was more frequent in primipara versus multipara, with an OR of 1.6. These findings are in line with previous research suggesting that the parity-dependent susceptibility for malaria in pregnancy is due to the development of immunity through infections in the previous pregnancy [9,18]. The positivity rates for malaria in our study population are alarmingly high and show that malaria is a major problem. Effective interventions to reduce malaria infections during pregnancy are the intermittent preventive treatment in pregnancy with IPTp-SP at every ANC visit from the second trimester onwards and the provision of bed nets. Results from the DHS show that the uptake IPTp-SP is inadequate, as only 44.4% of women in rural areas receive two or more doses during their ANC visits [2]. Ideally, pregnant women should receive three or more doses of SP, since reinfections with the two-dose regimen are common. Women who receive three or more doses of SP during their pregnancy gave birth to children with a higher mean birth weight and were less likely to have malaria at the time of delivery. Additionally, lower rates of anaemia were observed [24]. When taking into account the high rates of malaria found in our study and the low uptake of SP reported by the DHS, the existing interventions to reduce malaria—usage of bed nets and uptake of SP—should be evaluated and optimized.

In our study population, 10.4% of adult pregnant women were acutely undernourished, most likely as a consequence of food insecurity. These findings are in line with the DHS conducted in 2013, which found that, based on BMI, 10.2% of women (non-pregnant) in rural areas and Tonkolili district were acutely undernourished [2]. Since the rate of maternal malnutrition is high in our population and known to significantly contribute to adverse outcomes for both mothers and neonates, an intervention, such as a supplementary feeding program as introduced in other parts of Sierra Leone, would be justified.

Obesity has been on the rise in low- and middle-income countries for the past decades and, during pregnancy, obesity is a risk factor for developing gestational diabetes, preeclampsia and intrapartum complications such haemorrhage, caesarean delivery, infection and death [12]. The rate of obesity (5.9%) in our pregnant study population was roughly double the rate of obesity (2.9%) reported for women in rural areas by the DHS 2013 [2]. Reports have indicated that for the entire African region, levels of undernutrition have remained rather stable, while obesity is increasing [12]. Although the numbers in our study are small, a similar trend can be seen when comparing our data with the DHS 2013 findings. Unfortunately, the preliminary DHS report from 2019 does not report on the nutritional status. The increasing rate of obesity in developing countries is a result of a changing diet, with more sugar, fat, added meat and bigger portion sizes, less physical activity and higher social economic status [25].

A combined malnutrition (both under- and over-nutrition) prevalence of 16.3% suggests that the importance of nutritional programs providing information on and improved access to healthy nutrition cannot be overemphasised.

Anaemia was abundant, as 65.9% of all pregnant women in our study population suffered from low haemoglobin levels. This high prevalence is likely to be largely attributable to the high level of malaria infections, poor nutritional status and endemic soil-transmitted helminthiasis of our target population. Further research has also been done about the relationship between malaria and malnutrition, since a synergistic effect was suspected, but a large meta-analysis did not provide conclusive evidence [26].

Another important finding is the high proportion of adolescent pregnancies (16.4%) in the study population. This finding is in line with DHS data, indicating that the majority (44.9%) of 19-year-old women had already experienced childbearing [3]. Adolescents were more likely to present with malaria and anaemia at their first ANC visit, as reflected by the ORs of 2.1 and 1.7, respectively. This might be partially explained by the high number of primipara, who are more susceptible to malaria compared to multipara [9], in this group.

However, it should be noted that the percentage of pregnant adolescents corresponding to 16.4% was calculated on the basis of self-reported age. As many women are not sure of their exact age, there were large proportions of women reporting ages of 20, 25 and 30 years in our study population. This possibly caused an underestimation of the actual proportion of adolescents, as women (or girls) might simply round up their age to 20.

In the Sierra Leonean setting, pregnant adolescents suffer greatly from stigma and social abandonment. It is often thought that the lack of social support, rather than physical immaturity, is the main contributing factor for increased maternal deaths in this group [27]. This highlights the vulnerability of the adolescent group. The authors will explore the possibility to set up a framework for more social support for pregnant adolescents.

### Limitations

One of the main limitations of this study is a selection bias; by only using data from pregnant women attending the ANC clinic of a referral hospital, obviously many cases are left out. A rough calculation using birth rates suggested that approximately 50% of all pregnant women in our catchment area attend our ANC clinic at least once. Given the many difficulties in providing access to healthcare in this setting, the authors consider it quite an achievement to cover half of all pregnant women attending the HBVP. Most likely, the missing half will be pregnant women from remote areas limited by resources, who might also have constraints visiting their nearest health facility for routine antenatal care or seeking medical care once in labour. The presented results might therefore underreport the levels of maternal anaemia, malaria and malnutrition.

Another factor might be that women with complaints or with previous complicated pregnancies or births are more inclined to seek medical care, and therefore complicated pregnancies are likely to be over-represented in our population.

In the present study, only first ANC visits were included to reduce further over-representation of complicated cases, since only complicated cases would come back for follow-up, and uncomplicated cases are routinely sent back to their nearest PHU for subsequent ANC visits and delivery.

Another limitation could be the chosen time frame of the study and the wrongful representation of malaria incidence due to seasonal variations. To avoid this, a time frame was chosen that covered parts of both rainy and dry seasons. Additionally, no interventions relating to Mother and Child Health were introduced during this period. Lastly, due to the retrospective design of the study and the use of existing records, the authors had to rely on others for adequate recordkeeping.

## 5. Conclusions

Our gap analysis reveals a high burden of malaria, anaemia and malnutrition among pregnant women attending routine antenatal care at secondary healthcare level in rural Sierra Leone. Although prevention and treatment of these conditions are incorporated in all antenatal care protocols as recommended by the WHO, the current provision of care is clearly not sufficient to meet minimal needs. At the facility level, we have intensified counselling during the ANC visits in order to optimize adherence to the advised intake of iron and folic acid supplements, methods to prevent malaria and intake of a balanced diet. Clearly, a reduction of MMR will require more interventions addressing the quality of comprehensive ANC services on a national scale.

## Figures and Tables

**Table 1:** Basic characteristics of pregnant women attending their first antenatal care visit. EGA, estimated gestational age.

Characteristic	No of Cases = *N*	Average (Mean)	Lowest Value	Highest Value
Age	610	26 years	12	54
EGA	530	29 weeks	8	40

**Table 2:** Basic characteristics of pregnant women attending their first ANC visit. RDT, rapid diagnostic test, MUAC, mid-upper arm circumference.

Health Indicator	Percentage (%)
Adolescent pregnancy (<19 years)	16.4
Primipara	20.0
2nd–4th pregnancy	55.1
Grande multipara (>5 pregnancies)	24.9
Anaemia	
Total (Hb < 11.0 g/dL)	65.9
Mild (Hb 10.0–10.9 g/dL)	30.1
Moderate (Hb 7.0–9.9 g/dL)	35.6
Severe (Hb < 7.0 g/dL)	0.2
Anaemic adolescents	75.6
Anaemic adults	63.9
Malaria positivity rate (RDT for *Plasmodium falciparum*)	
Total	35.2
Primipara	44.6
Multipara	32.9
Adolescents	50.6
Adults	32.4
Other infections	
HIV-positive	0.8
Syphilis positive	0.2
Malnutrition	
Undernourished (MUAC < 23)	10.4
Overnourished (MUAC > 30)	5.9

**Table 3:** Positivity rates of RDTs (*P. falciparum*) in pregnant women, routinely tested during ANC.

Month	Total RDTs Done at the ANC Clinic	Positive	Positivity Rate (%)
August	160	77	48.1
September	85	29	34.1
October	131	46	35.1
November	70	16	22.9
December	79	17	21.5
